# Target-Specific Machine Learning Scoring Function Improved Structure-Based Virtual Screening Performance for SARS-CoV-2 Drugs Development

**DOI:** 10.3390/ijms231911003

**Published:** 2022-09-20

**Authors:** Muhammad Tahir ul Qamar, Xi-Tong Zhu, Ling-Ling Chen, Laila Alhussain, Maha A. Alshiekheid, Abdulrahman Theyab, Mohammad Algahtani

**Affiliations:** 1 State Key Laboratory for Conservation and Utilization of Subtropical Agro-Bioresources, College of Life Science and Technology, Guangxi University, Nanning 530004, China; 2 National Key Laboratory of Crop Genetic Improvement, Hubei Key Laboratory of Agricultural Bioinformatics, College of Informatics, Huazhong Agricultural University, Wuhan 430070, China; 3 Department of Biology, College of Science, Qassim University, Buraydah 51452, Saudi Arabia; 4 Department of Botany & Microbiology, College of Science, King Saud University, Riyadh 11451, Saudi Arabia; 5 Department of Laboratory & Blood Bank, Security Forces Hospital, P.O. Box 14799, Mecca 21955, Saudi Arabia; 6 College of Medicine, Al-Faisal University, P.O. Box 50927, Riyadh 11533, Saudi Arabia

**Keywords:** machine learning, target specific scoring function, smina, SARS-CoV-2, COVID-19

## Abstract

Leveraging machine learning has been shown to improve the accuracy of structure-based virtual screening. Furthermore, a tremendous amount of empirical data is publicly available, which further enhances the performance of the machine learning approach. In this proof-of-concept study, the 3CL^pro^ enzyme of SARS-CoV-2 was used. Structure-based virtual screening relies heavily on scoring functions. It is widely accepted that target-specific scoring functions may perform more effectively than universal scoring functions in real-world drug research and development processes. It would be beneficial to drug discovery to develop a method that can effectively build target-specific scoring functions. In the current study, the bindingDB database was used to retrieve experimental data. Smina was utilized to generate protein-ligand complexes for the extraction of InteractionFingerPrint (IFP) and SimpleInteractionFingerPrint SIFP fingerprints via the open drug discovery tool (oddt). The present study found that randomforestClassifier and randomforestRegressor performed well when used with the above fingerprints along the Molecular ACCess System (MACCS), Extended Connectivity Fingerprint (ECFP4), and ECFP6. It was found that the area under the precision-recall curve was 0.80, which is considered a satisfactory level of accuracy. In addition, our enrichment factor analysis indicated that our trained scoring function ranked molecules correctly compared to smina’s generic scoring function. Further molecular dynamics simulations indicated that the top-ranked molecules identified by our developed scoring function were highly stable in the active site, supporting the validity of our developed process. This research may provide a template for developing target-specific scoring functions against specific enzyme targets.

## 1. Introduction

The COVID-19 pandemic originated from a continuously evolving novel type of beta coronavirus, SARS-CoV-2 or 2019-nCoV, which has shaken the global population [1,2]. COVID-19, the disease caused by SARS-CoV-2, is similar to those caused by coronavirus outbreaks in recent years, such as SARS-CoV in 2003 and MERS in 2012 [3,4]. It is characterized by respiratory symptoms such as fever, dry cough, fatigue, and loss of taste or smell. In more severe cases, COVID-19 can cause pneumonia, dyspnea, and death [5,6]. Globally, scientists have increased their efforts to develop and/or identify potential targets for refurbishing and repurposing drugs to find possible cures and drugs. Pharma companies are currently developing vaccines that offer adequate protection against virus spread and harm [7]. The success of vaccination campaigns worldwide has been compromised by the identification of new mutations. Therefore, drug development/refurbishment to combat virus replication and cell entry protocol can be used to slow down or deactivate virus replication and cell entry. The in silico approach appears to be an efficient method to narrow down an extensive collection of compound choices to a few tens or hundreds of compounds that could block the active site of a particular protein.

The SARS-CoV-2 virus belongs to the single-stranded positive-sense RNA family. There are four structural proteins encoded by this virus family—a small envelope protein (E), a matrix protein (M), a nucleocapsid phosphoprotein (N), and a spike protein (S)—and 16 nonstructural proteins (nsp1–16), which work together to ensure replication in the host cell [8]. Viral replication is accomplished by nonstructural proteins that carry out enzymatic functions. In addition to nsp7, nsp8, and nsp12, which all form the RNA-dependent RNA polymerase complex, the SARS-CoV-2 genome also encodes proteases nsp3, 3CL^pro^, and nsp5, which inhibit innate immunity, as well as a protein called NPEP, which breaks down viral polyproteins [9,10]. 

**Figure 1:**
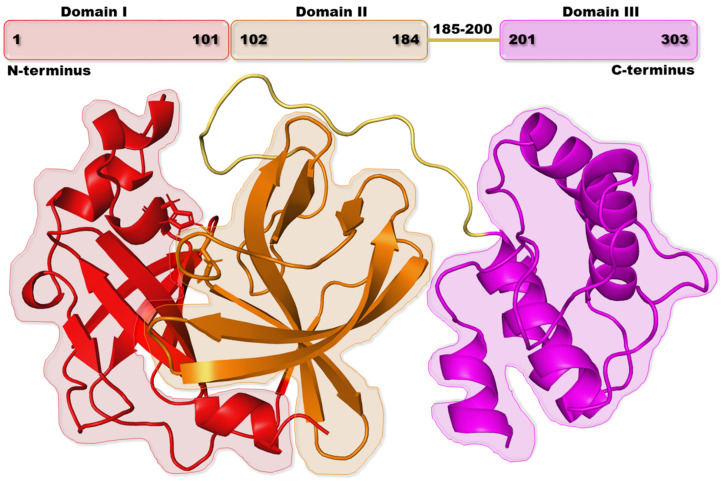
Domain organization and structural view of the 3CL^pro^ enzyme.

Other important factors that control the spread of a virus in the human body include: (1) the entry of the virus into cells, (2) inhibition of polyprotein proteolysis that produces new virions, and (3) replication of the RNA genome. Therefore, possible treatment targets for SARS-CoV-2 would be (1) SARS-CoV-2 receptor-binding spike proteins, (2) proteases 3CL^pro^, and (3) the RNA polymerase (RdRP) [11,12]. Angiotensin-converting enzyme 2 (ACE2) in humans serves as an entry receptor for SARS-CoV-2 spike proteins. Having knowledge of the interface between spike protein and ACE2 complex could be advantageous for vaccine development. Since proteases display high genomic homology (82–96%) and present inhibitory options for SARS-CoV-2 polyprotein proteolysis, they are attractive inhibitory targets [13]. The 3CL^pro^ monomer consists of three domains (domain I has residues 8–101, domain II has residues 102–184, and domain III has residues 201–303) and a six-residue loop (residues 185–200) that connects domains II and III (Figure 1).

Computer-aided drug discovery is currently one of the fastest-growing topics in machine learning [14]. In contrast to simulations based on explicit physical equations, machine learning approaches identify relationships between empirical observations of small molecules and extrapolate those relationships to predict the chemical, biological, and physical properties of novel compounds [15]. Machine learning has been primarily used in drug discovery to provide researchers with a better understanding and exploitation of chemical structures and their biological activities [16]. A hit compound from a drug screening campaign might require optimization of its chemical structure. This will improve its binding affinity, biochemical properties, or biological responses. The solution to this type of problem used to be labor-intensive, time-consuming, and expensive in medicinal chemistry. In today’s modern world, artificial brains can accurately predict in silico how chemical modifications influence biological behavior using advanced machine learning techniques such as quantitative structure-activity relationships (QSAR) or quantitative structure-property relationships (QSPR) [17,18]. 

**Figure 2:**
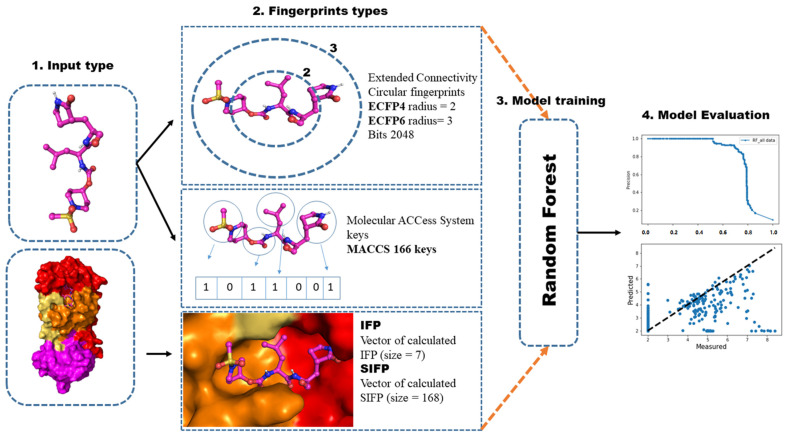
Workflow of 3CL^pro^-specific machine learning scoring function. The input was docked poses of proteins and ligands in the pdb and mol2 format.

There are three basic types of traditional scoring functions for scoring and ranking protein-ligand complexes: force field-based, knowledge-based, and empirical [19]. Among the methods for building scoring functions, machine learning and deep learning have shown great success for a long time. Most recently, convolutional neural networks used structural information of proteins-ligand complexes to predict binding affinity and conduct virtual screenings [20,21]. Further, a medicinal chemist usually only focuses on one target at a time and attempts to use the scoring function that provides the best result. Target-specific scoring functions (TSSF) for each target are the most common and direct way of addressing this issue. The present study used smina as a baseline scoring function. In contrast to AutoDock Vina, smina provides enhanced minimization and scoring capabilities. Smina is available under a GPL2 license at http://smina.sf.net (accessed on 15 June 2022). We compared smina, a generic classical scoring function, with our developed target-specific scoring function. Typically, smina is effective when there is a linear relationship between features and potency, which is very rare in many instances. Alternatively, machine learning-based scoring functions exploit their nonlinearity and can be applied to both linear and nonlinear relationships. There are only a few interaction terms used by smina, such as Gaussian terms, repulsion terms, hydrogen bonds, and hydrophobic terms, which comprise the default scoring function, a non-hydrophobic contact term, and a Lennard-Jones 4–8 van der Waals term. Using machine-learning techniques, we developed a pipeline that leads to developing a 3CL^pro^-specific scoring function (Figure 2). We used a random forest classifier to predict molecule class and random forest regression to determine the molecule’s activity [22,23]. Molecular dynamics simulations were utilized to validate the results. The present study will have prospective applications for structure-based virtual screening against the 3CL^pro^ enzyme of SARS-CoV-2. 

## 2. Results and Discussion

### 2.1. Chemical Space Analysis

**Figure 3:**
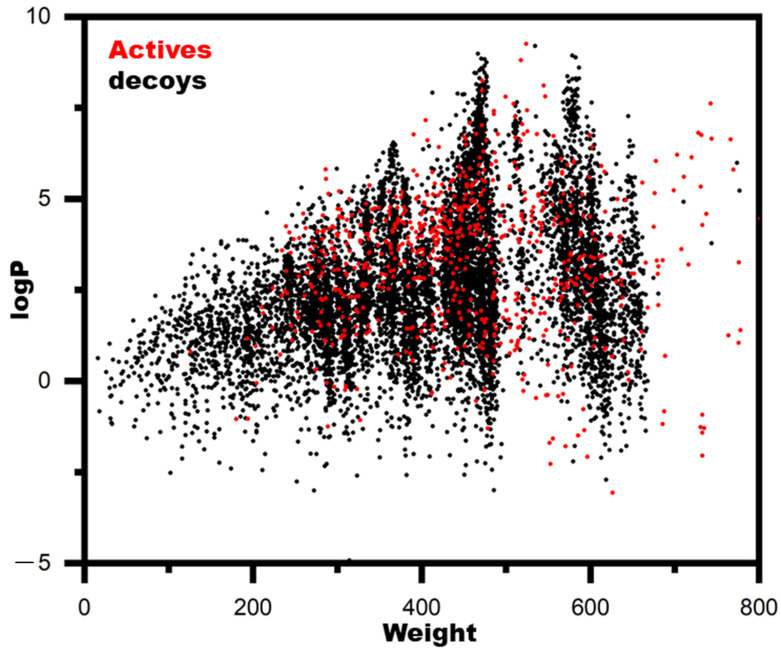
Chemical space analysis of the actives and decoys. The chemical space was defined as the weight and logP.

Model performance is dependent on the chemical diversity of molecules in the active and decoy sets. One objective of this study was to identify the class of molecules. BindingDB was used to collect all active molecules, and DeepCoy was used to generate decoys. Then, the chemical space analysis of the actives and decoys was conducted (Figure 3). The chemical space was defined as the weight and logP of the molecules. Actives and decoys were equally disturbed.

**Figure 4:**
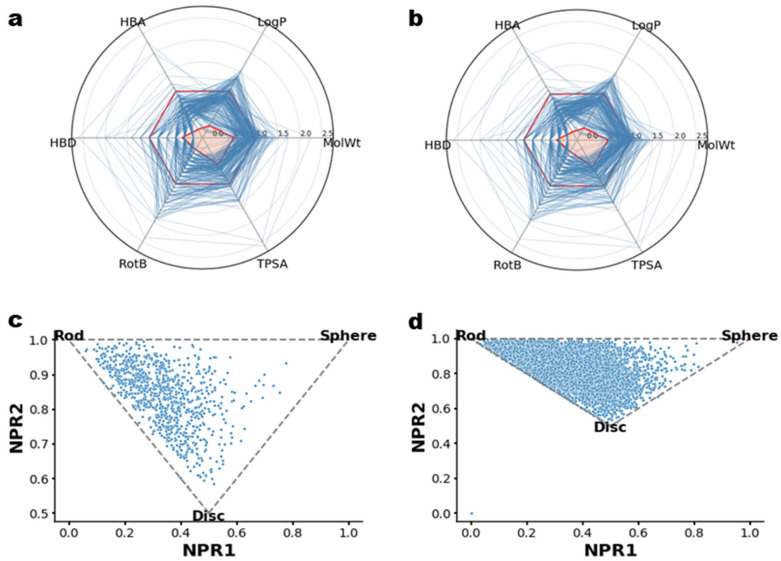
Characteristics of the actives and decoys molecules. Lipinski’s Rule of Five (Ro5) analysis of (**a**) active and (**b**) decoys molecules. Normalized principal moments ratio (NPR) analysis of (**c**) actives and (**d**) decoys.

Furthermore, Lipinski’s Rule of Five analysis (Ro5) was also performed to remove any bias from the data. According to the results, actives and decoys had the same physicochemical properties, thus validating the DeepCoy algorithm’s ability to produce decoys with the same physiochemical properties. The 3D structural arrangement was also checked in terms of shape distribution. The molecular shape distribution of compounds was assessed using a normalized principal moments ratio (NPR) analysis. Based on the results, the minimum energy conformers of the compounds from all actives and decoys molecules presented approximately the same shape, with rod- and disk-shaped compounds dominating and only a few molecules exhibiting a round shape. According to the chemical space analysis, both actives and decoys possessed the same physiological properties but different structural characteristics, which positively influenced the random forest algorithm performance (Figure 4).

### 2.2. CL^pro^-Specific Scoring Training

**Figure 5:**
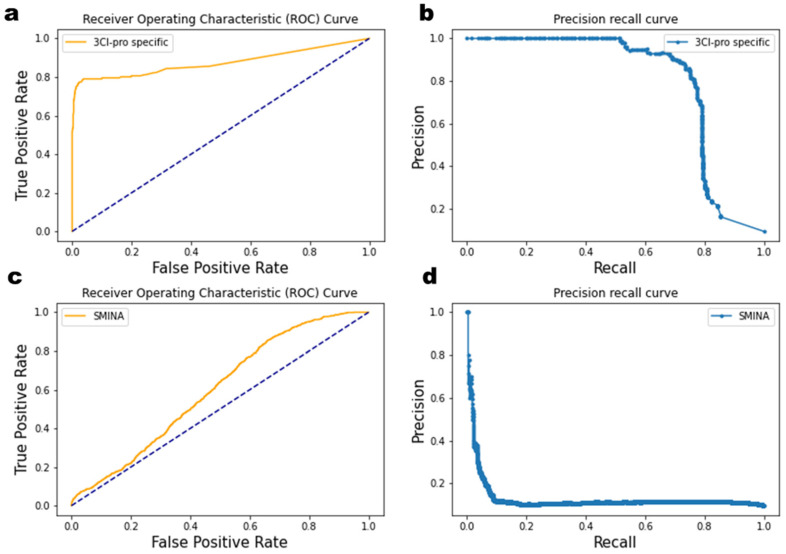
Target specific scoring function performance: (**a**,**c**) ROC curve, and (**b**,**d**) precision-recall curve.

Random forest classifiers and regressor algorithms were trained in the present study. Two types of fingerprints were used for model training. The chemical structure is reflected in the first type of fingerprint, ECFP4, ECFP6, and MACCS. The second type of fingerprint, the IFP and SIFP, reflects the interaction between a protein and its ligand. The length of ECFP4 and ECFP6 was 2048 bits, while MACCS had a length of 166 bits. There were 7 bits in the IFP and 168 bits in the SIFP. All these features were merged, and training was carried out with 80% of the data. The remaining 20% of data was used to test the algorithm performance. The maximum performance was noted at n_estimators = 500, max_features = ‘sqrt’ parameters. The ROC of the model did not show good results because the data were not balanced (902 actives, 9020 decoys) (Figure 5a). A precision-recall curve is the best tool for unbalanced data. The area under the precision-recall curve was found to be 0.80 (Figure 5b). The performance of the 3CL^pro^-specific function was compared to the inbuilt scoring function of smina software. Compared to the ROC of 3CL^pro^ specific, the ROC curve of the smina scoring function was far away from the right corner, implying that the smina scoring function did not perform well because smina uses generic scoring (Figure 5c). Similarly, the area under the precision-recall curve for smina scoring was found to be 0.13, which is not considered a good performance. From the above results, the machine learning model trained for a specific enzyme performed well as compared to the generic scoring function. 

### 2.3. Enrichment Factor Analysis

**Figure 6:**
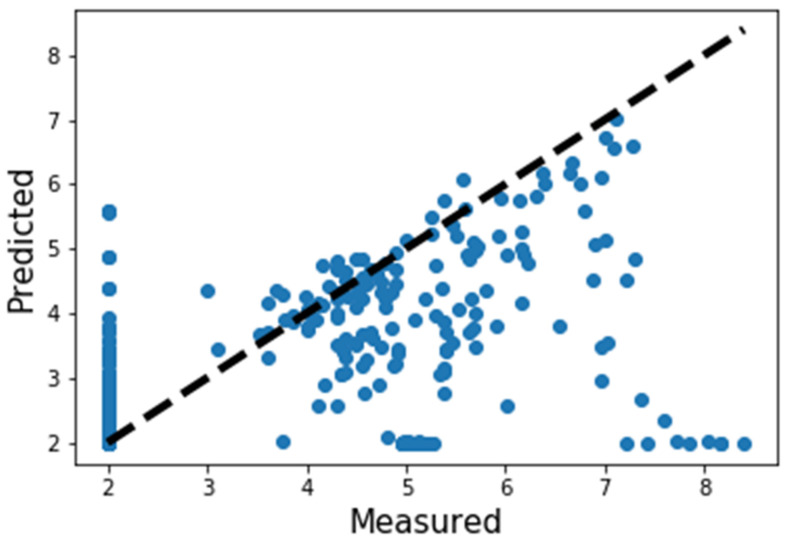
Correlation graph of actual and predicted pIC50 values.

Our developed scoring function, *RandomforestRegressor(),* showed the best performance in predicting and ranking the actives molecules. The evaluation was carried out in terms of enrichment factor (EF%) analysis at a different percentage. By definition, the enrichment factor is calculated by comparing the number of active compounds found in the top 1% of a ranked compound library with the number of active compounds expected by random selection. The proportion of actives found within the top 1% of ranked libraries is measured as the hit rate in the top 1% (HR1%). EF1% is defined as HR1% divided by HR100%, with the latter corresponding to the proportion of actives within the full library. For the smina score, the enrichment factors at EF1%, EF3%, and EF5% were found to be 1.3, 1.4, and 2.2, respectively. For our developed scoring function, the EF1%, EF3%, and EF5% were found to be 2.3, 3.1, and 3.4, respectively. A good correlation was also found between the predicted and actual pIC50 values (Figure 6). 

The actual pIC50 value of the top five molecules based on the smina score was compared to the pIC50 of the top five molecules based on our developed scoring function (Table 1). Our developed scoring had better ranking ability than the smina score. There were two decoy molecules, mol_1170 and mol_1112, wrongly scored by the smina in the top five molecules. The top two molecules, mol_336 and mol_821, were subjected to MD simulation to check their stability to confirm the validity of our developed pipeline. 

### 2.4. Stability of Top-Ranked Molecules

**Figure 7:**
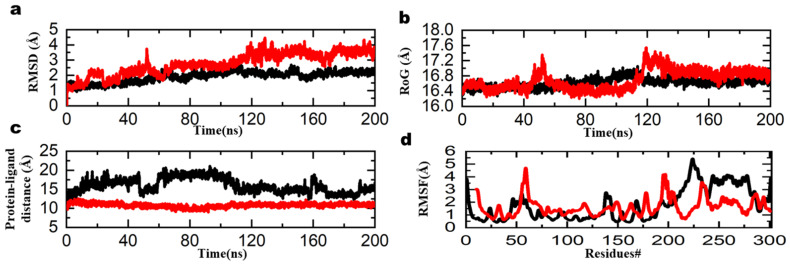
Dynamics stability of the top 2 molecules: (**a**) RMSD, (**b**) RoG, (**c**) protein-ligand distance, (**d**) RMSF.

To verify the stability of the top two molecules, a molecular dynamics simulation was performed. Simulations were performed for 200 ns on each system. The stability of each system was evaluated by measuring the root mean square deviation (RMSD) (Figure 7).

**Figure 8:**
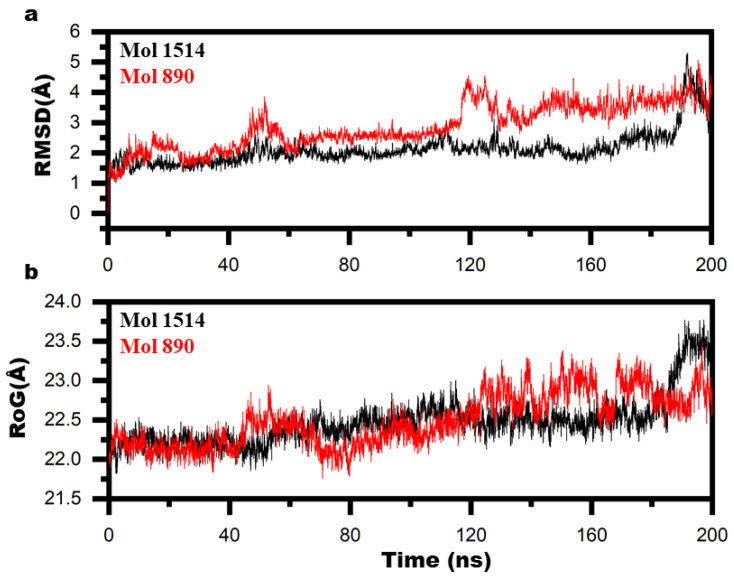
Dynamis stability of the top 2 molecules screened with smina: (**a**) RMSD, (**b**) RoG.

According to the RMSD graph, the two molecules were highly stable in the active state (Figure 7a). Based on the MMGBSA results, there is also evidence of the stability of the molecules with binding energies of −93.54 kcal/mol and −84 kcal/mol for mol_336 and mol_821, respectively. In addition, the stability of the interaction is confirmed by the average distance between the protein and the ligand (Figure 7c). The graph illustrates that the ligands remained in the active site throughout the molecular dynamics simulation. The local fluctuation plot shows that all motifs remained stable (Figure 7d). The protein structure remained compact and stable (Figure 7b). These results show that two molecules were highly stable and were top ranked by our developed scoring function. On the other hand, we also simulated the top two molecules ranked by smina. Their RMSD graph and radius of gyration showed unstable pattern, owing to the instability of the two molecules in the active site (Figure 8). Their MMGBSA also confirmed the weaker binding of these two molecules as shown by their values (−64 kcal/mol and −70 kcal/mol). These results suggested that our developed scoring function retrieved potent molecules compared to the generic scoring function of smina. 

## 3. Materials and Methods

### 3.1. Preparation of Actives

In total, 1170 compounds in BindingDB databases showed experimental activity against the human 3CL^pro^ and were retrieved (www.bindingdb.org; accessed on 10 May 2022). We excluded compounds with missing IC50 values from the datasets. Duplicate molecules were removed from the database based on the SMILE notation. As a result of the above preprocessing, the BindingDB datasets consisted of 902 compounds. Following that, the IC50 for each dataset was converted to pIC50 using the following equation:

$$\mathrm{pIC}50=-\log(\mathrm{IC}50\left(M\right))$$

where IC50 was first converted to molar unit M from µM and nM. 

### 3.2. Preparation of Decoys

**Figure 9:**
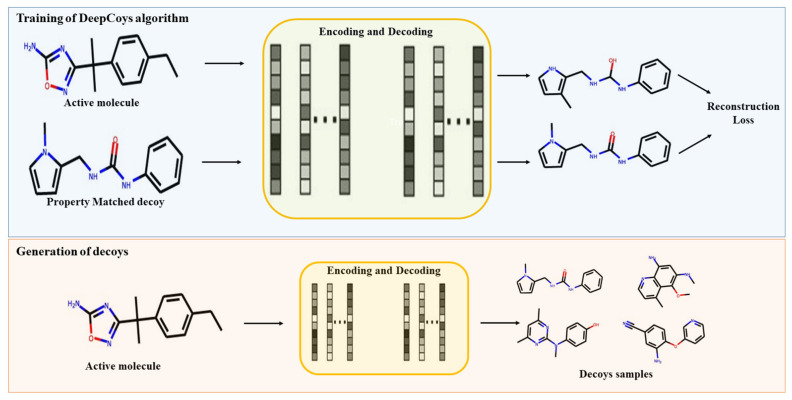
Decoys generation process through DeepCoys algorithm. The figure was adapted from [24].

Graph-generative neural networks were used to generate property-matched decoys with the DeepCoy algorithm, which works on the basis of linking algorithms [24]. The input for this method is an active compound, which is used to generate molecules with structurally different properties yet similar physiochemical properties (Figure 9). New molecules are built by iteratively building them atom-by-atom from a pool of atoms. In addition to the atomic valency rules that ensure the molecules are chemically valid, DeepCoy also incorporates a minimal amount of chemical knowledge. This takes the form of a defined set of atom types and basic atom valency rules. The generated models construct decoys with properties similar to the graphs of active molecules using a standard gated-graph neural network in both the encoder and decoder. In total, 100 decoys were generated for each compound, and 10 out of 100 optimized decoys were selected. 

### 3.3. Generate 3D Coordinates for Actives and Decoys

Using the Pybel package, we converted the smiles generated from actives and decoys into 3D coordinates [25]. The Pybel library is the Python wrapper for the OpenBabel cheminformatics toolkit. The make3D function of Pybel generated the 3D coordinates using mmff94s forcefield in 50 steps. The localopt function was used to optimize the geometries further using the same forcefield with 500 steps. All 3D geometries were saved in mol2 format.

### 3.4. Molecular Docking

Docking was carried out using the Smina software [26]. The following parameters were used while running the docking code of Smina: the number of restarts for conformational searching (--exhaustiveness = 8) and the number of poses to output for each docked molecule. Molecular docking typically involves building a user-defined docking space and exploring possible ligand binding conformations within it [27]. The Python package *getbox()* was used for generating the docking grid box. The experimental structure of 3CL^pro^ with PDB ID 6LU7 was retrieved from the PDB databank and used for docking [28]. The output docked poses were saved in SDF format. 

### 3.5. Generation of Descriptors

The molecular structure of every compound in the dataset was represented by physical and chemical descriptors and molecular fingerprints. Using the rdkit, MACCS fingerprints, ECFP4 fingerprints, and ECFP6 were calculated to characterize the physicochemical properties, chemical structures, and drug-like properties of the investigated compounds. For protein-ligand interaction fingerprints, the docked protein-ligand complexes were used, and IFP and SIFP were generated using the Open Drug Discovery Toolkit (oddt) [29]. Oddt is a cheminformatics Python library for computer-aided drug designing. MACCS fingerprints have 166 binary fingerprints as substructure keys, each of which indicates the presence of 1 of the 166 substructures [30]. ECFP4 and ECFP6 are circular topological fingerprints with 2048 descriptors [31]. There are 7 bits in the IFP and 168 bits in the SIFP representing hydrophobic contacts, aromatic face-to-face, aromatic edge-to-face, hydrogen bond (protein as a hydrogen bond donor), hydrogen bond (protein as a hydrogen bond acceptor), and salt bridges (protein positively charged, protein negatively charged, and ionic bond with metal ion). IFP represent the presence and absence of each interaction and return a vector of size = 8, while SIFP return a matrix of vector size = 168, expressing the presence and absence of each all interactions for each of the 20 amino acids. All features were concentrated to form single vector of size = 4438 and used as input for model training. 

### 3.6. Scoring Function

For the scoring function, the random forest algorithm was used. The *RandomforestClassifier()* function was used to predict the molecule class, while *RandomforestResgressor()* was used to predict the pIC50 of the molecules. Several n_estimators (10, 20, 50, 100, 1000) were used to optimize the algorithm for better performance. 

### 3.7. Model Evaluation

An area under the receiver operating characteristic curve (ROC) is preferred for virtual screening because it is robust and does not require user-defined parameters [32]. It plots the relationship between true positive rate (TPR, also called recall or sensitivity) and false-positive rate (FPR, equivalent to 1-specificity), defined by the equation below:

TPR=TPTP+FN

FPR=FPFP+TN

An alternative metric is an area under the precision-recall curve (AUC[PR]) [33]. In virtual screening, AUC[PR] summarizes classifier performance better than AUC[ROC] when the class labels are highly skewed or unbalanced. The reason is that there are usually few active compounds present in the dataset compared to inactive ones. AUC[PR] examines a classifier’s ability to discover actives (recall) and whether predictions are correctly classified (precision) at different prediction thresholds. The precision and recall are defined by the following equation:

Recall=TPTP+FN

Precision=TPTP+FP

Virtual screening metrics can also be measured using enrichment factors (EF) proportional to the number of actives present in the prioritized subset of compounds compared to the expected number of actives in a subset drawn randomly [34]. Virtual screening represents the number of active compounds found in the top 1% of ranked compound libraries compared to the number of compounds found through random selection. The hit rate in the top 1% (HR1%) is the proportion of actives found in the top 1%. By definition, EF1% is HR1% divided by HR100%, the latter corresponding to the proportion of actives in the full library. The following equations define EF1%:

EF1%=HR1%HR100%

$$HR1\%=\frac{Number\;of\;actives\;found\;in\;the\;top\;1\%\;of\;total\;ranked\;compounds}{Number\;of\;total\;compounds\;in\;the\;top\;1\%\;}$$

$$HR100\%=\frac{Number\;of\;actives\;found\;the\;whole\;dataset}{Number\;of\;total\;compounds\;in\;the\;dataset}$$

### 3.8. Molecular Dynamics (MD) Simulation

The stability of top scored molecules was evaluated through MD simulation using amber20 code with ambertool21 [35]. The ff19SB force field was used to define protein. For ligand topology, an antechamber was used. Each system was solvated in a rectangular box of the OPC water model. The size of the water box was chosen according to each complex’s size to balance speed and accuracy with 12 and 8 buffer distances, respectively. Each system was neutralized with Cl- ions. The steepest descent and conjugate gradient techniques were used to relax each system to remove bad clashes between atoms. Subsequently, each system was heated up to 300 K. After heating, each system was equilibrated by a two-step procedure at constant 1 atm and 300 K. First, we equilibrated the density with weak restraint for 2 ns. Second, we equilibrated the system without any restraint for more than 2 ns. Finally, each system was subjected to a long-run production simulation. The Langevin thermostat controlled the temperature of each system [36]. The long-range electrostatic interactions were treated with the Particle Mesh Ewald algorithm [37]. The covalent bonds were treated with the SHAKE algorithm [38]. The GPU-supported pmemd code performed the production step of molecular dynamics simulation for each system [39], and the cpptraj package and g_sham module of gromacs were used to analyze the trajectories.

## 4. Conclusions

The present study utilized the machine learning approach to set up a pipeline that scores and ranks biological molecules against the 3CL^pro^ enzymes. All experimental data were obtained from the bindingDB database. We showed that the random forest trained on MACSS, ECFP4, ECFP6, IFP, and SIFP correctly identified the molecule’s class and activity. Our developed model will have prospective applications for structured-based virtual screening against 3CL^pro^ of SARS-CoV-2. Moreover, we compared the performance of the 3CL^pro^-specific scoring function with smina generic scoring. Our study shows that target-specific machine learning scoring function has better performance compared to the classical generic scoring function. Since machine learning tasks depend on experimental data, the model performance will be further enhanced and will include more experimental observations in the future, as many groups are actively working on SARS-CoV-2 treatment. This study may serve as a template for developing target-specific scoring functions against specific enzyme targets.

## Figures and Tables

**Table 1:** Comparison of top 5 molecules ranked by Smina and our developed scoring function.

Top 5 Molecules Scored by Smina			Top 5 Molecules Scored by Smina 3CL^pro^-Specific Machine Learning Model		
Molecules Top 5	Smina Score	Actual pIC50	Molecules Top 5	3CL^pro^-Specific Score	Actual pIC50
Mol_1514	−10.80	4.79	Mol_336	6.95	7.10
Mol_890	−10.64	4.79	Mol_821	6.67	7.01
Mol_1170	−10.43	2	Mol_522	6.62	7.08
Mol_1112	−10.35	2	Mol_1355	6.47	7.27
Mol_280	−10.25	4.49	Mol_819	6.39	6.66

## Data Availability

The data presented in this study are available within the article.
